# Diode Laser Frenectomy: A Torch of Freedom for Ankyloglossia

**DOI:** 10.7759/cureus.58319

**Published:** 2024-04-15

**Authors:** Manisha Verma, Mohammad Abdurrahman Khan, Aehad Ul Haque, Syed Fiza Mustaqueem

**Affiliations:** 1 Department of Periodontology, King George's Medical University, Lucknow, IND; 2 Department of Forensic Medicine and Toxicology, Hind Institute of Medical Sciences, Lucknow, IND; 3 Department of Pathology, Integral Institute of Medical Sciences & Research, Lucknow, IND

**Keywords:** carbon dioxide laser, hypertrophic tissue frenulum, diode laser, lingual frenectomy, ankyloglossia

## Abstract

Ankyloglossia, also known as tongue-tie, is a rare congenital anomaly of the oral cavity that not only causes difficulties in breastfeeding and teeth cleaning but also causes difficulty in speech articulation. Our patient faced difficulty in freely moving his tongue because of the short lingual frenulum wherein laser lingual frenectomy was indicated. The patient was treated successfully with a soft tissue diode laser having a wavelength of 445 nanometers. The use of a low-wavelength diode laser becomes relatively complimentary to standard scalpel surgery because of patient consolation, offers a blood-free area, reduces inflammation and edema, and is less damaging to thermal tissues.

## Introduction

The tongue is an important oral structure that accomplishes speech, position of teeth, periodontal tissues, nutrition, swallowing, nursing, and certain social activities. The lingual frenulum is a thick fold of mucous membrane that runs from the floor of the mouth to the midline of the undersurface of the tongue. Frenulum based on tissue composition can be divided into three types: (1) fibrous frenulum, (2) muscular frenulum, and (3) mixed or fibromuscular frenulum. Ankyloglossia (tongue-tie), is an anatomical disorder, wherein the frenulum becomes too short and restricts the full protrusion of the tip of the tongue [1-3]. Ankyloglossia cases are more frequently found in men than in women [3]. Verbal articulation is obstructed, and the phonetic function is hindered and is especially noticeable with the letters t, d, n, l, s, r, and z. Further disorder resulted in atypical swallowing and incorrect support of the tongue, as ankyloglossia hinders the tongue from resting at the roof of the mouth [2]. Many untreated cases of ankyloglossia or advanced severe cases also result in several dental problems such as misaligned lower anterior teeth and abnormal development of the mandible [2-4]. Some patients present with narrow maxillary arch because of the lack of transverse growth, which results in crossbite. In other cases, ankyloglossia can also be present along with anterior open bite because of the low position of the tongue. The use of a low-power diode laser is effective in the treatment of ankyloglossia, reduction of edema, and postoperative healing. Thus, a lower wavelength of diode laser 445-450 nanometers (nm) can precisely incise and causes ablation of the oral soft tissues with minimal thermal damage [5]. This attribute of diode lasers helps provide a comparatively bloodless field and excellent postsurgical healing even without suturing the surgical area [6].

## Case presentation

**Figure 1:**
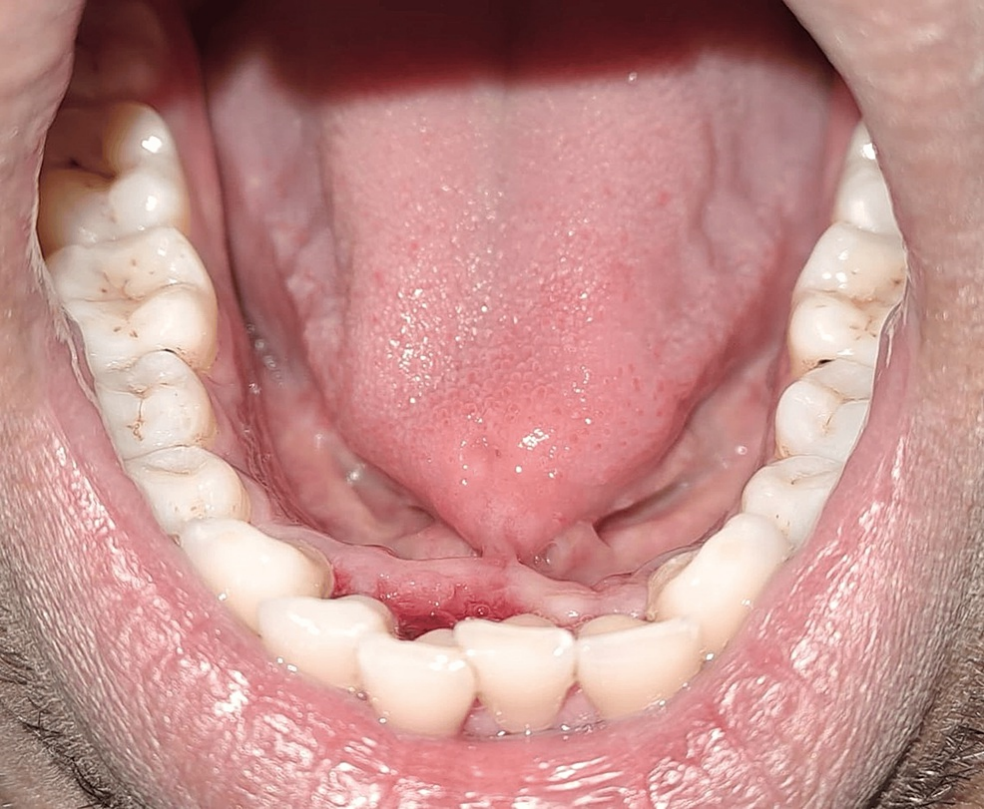
Front view of the tongue-tie

**Figure 2:**
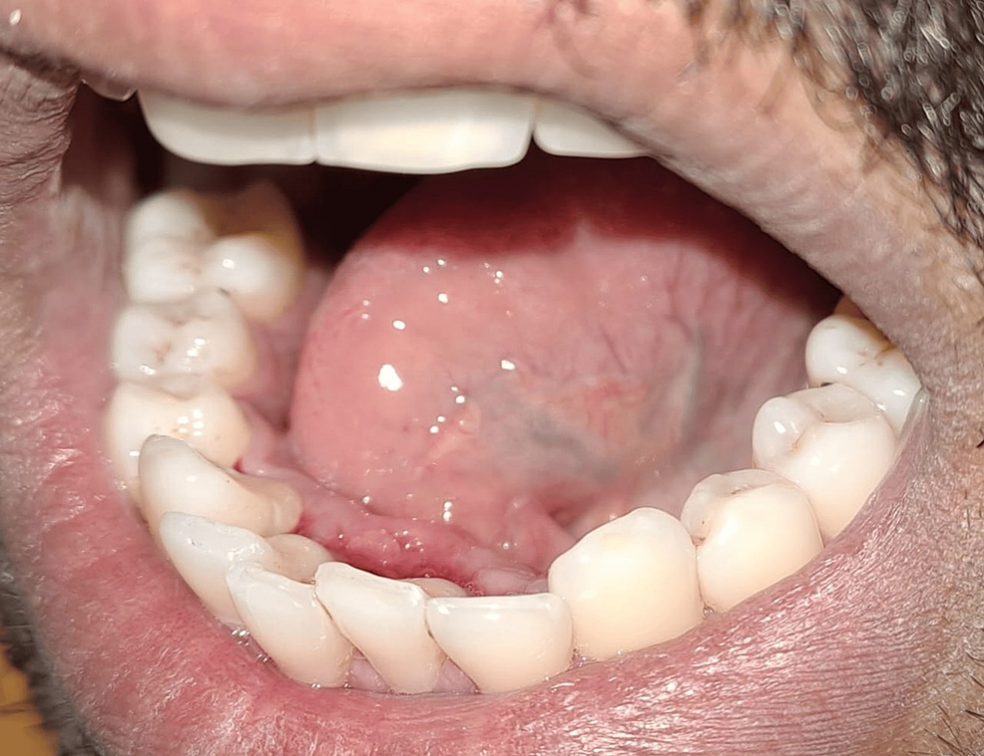
Left lateral view of the tongue-tie

**Figure 3:**
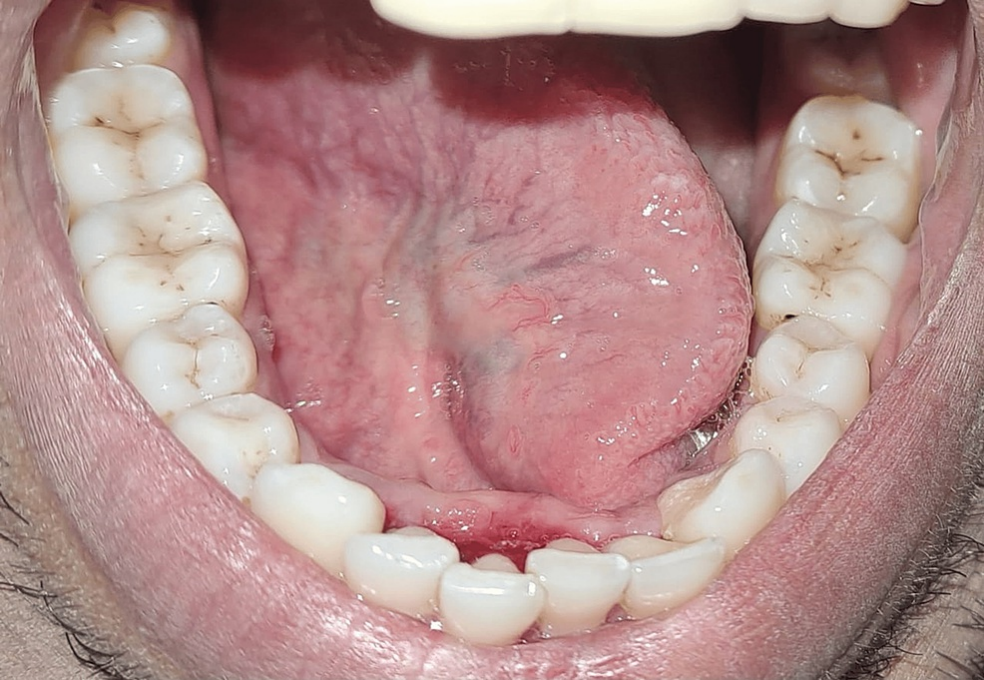
Right lateral view of the tongue-tie

A 39-year-old male patient came to our department with a chief complaint of difficulties in pronunciation with limited tongue movement, pain, swelling, and bleeding gums at the anterior aspect of the lower jaw. On clinical examination, the patient was asked to protrude his tongue, which showed restrictions in the tongue's movements (Figures 1-3).

**Figure 4:**
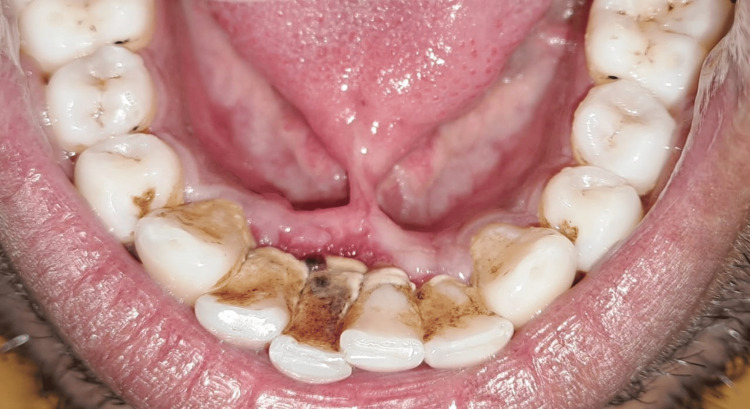
Ankyloglossia occlusal view before oral prophylaxis

The patient had poor oral hygiene because of the above-mentioned chief complaints. Intraoral examination revealed generalized chronic marginal gingivitis along with a thick lingual frenulum attached to the tongue tip (Figure 4).

After a clinical examination, a diagnosis of class III ankyloglossia (tongue-tie) was made according to Kotlow’s classification.

Kotlow’s classification:

Class I: Mild ankyloglossia (tongue-tie) with movement of the tongue of 12-16 mm.

Class II: Moderate ankyloglossia (tongue-tie) with movement of the tongue of 8-11 mm.

Class III: Severe ankyloglossia (tongue-tie) with movement of the tongue of 3-7 mm.

Class IV: Complete ankyloglossia (tongue-tie) with movement of the tongue <3 mm.

**Figure 5:**
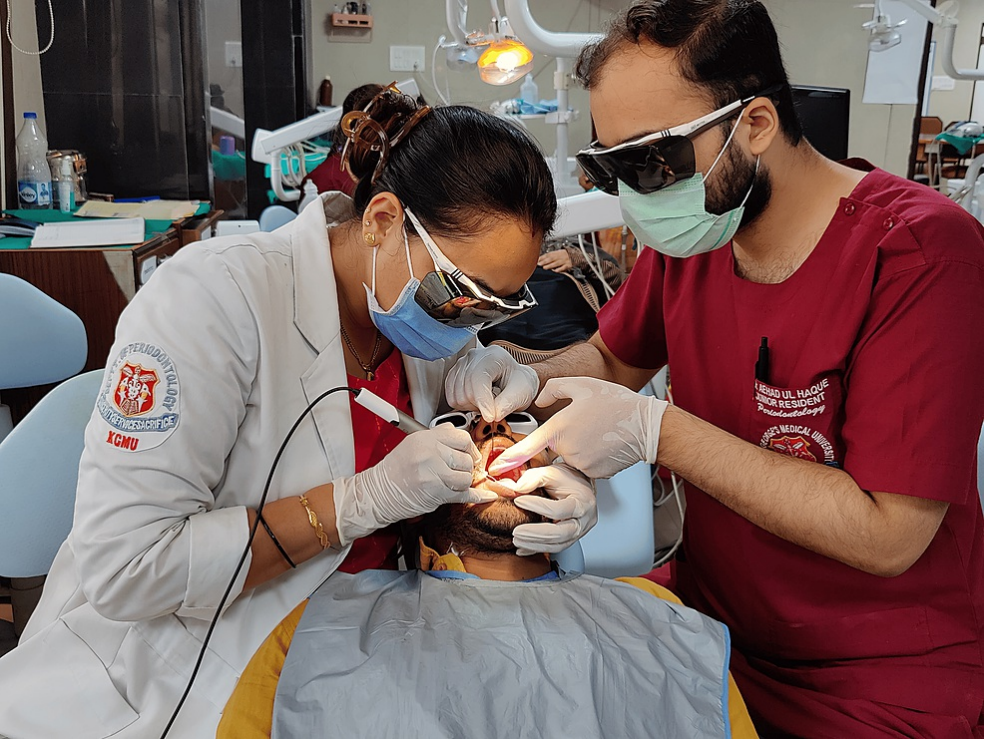
Performing laser frenectomy

The patient was advised for laser lingual frenectomy (Figure 5).

**Figure 6:**
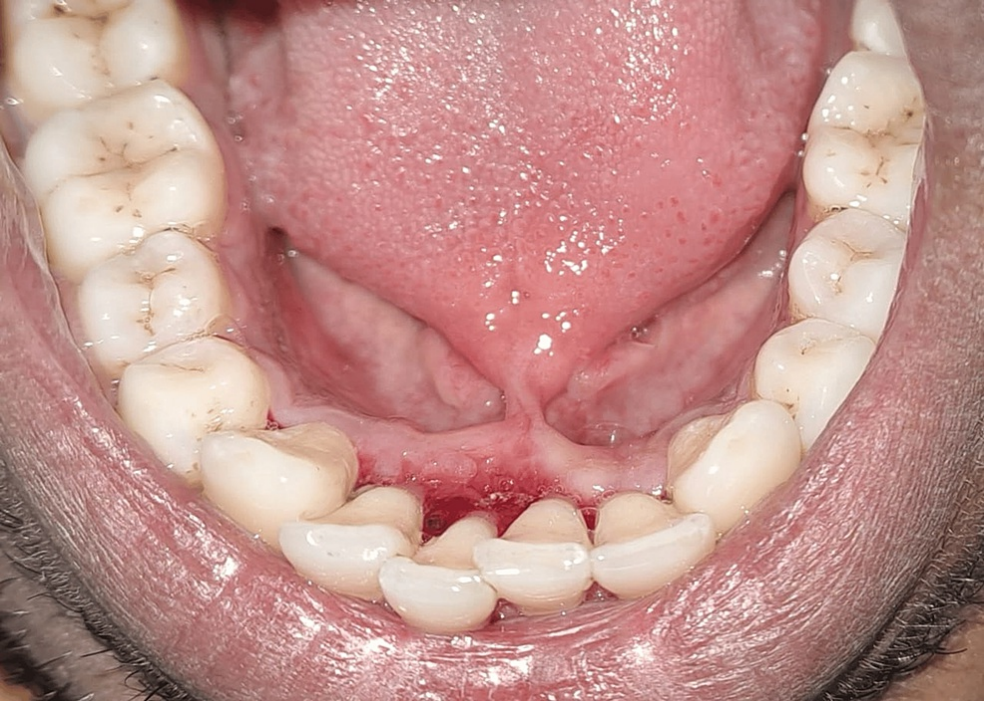
Ankyloglossia occlusal view after oral prophylaxis

A written informed consent was taken from the patient before the procedure of soft tissue diode laser lingual frenectomy. The patient was asked for the required investigation of blood after completion of phase 1 therapy (Figure 6).

Lingual frenectomy was performed with the help of a soft tissue diode laser. Before performing the procedure, bilateral local anesthesia (2% lignocaine and 1:80,000 adrenaline) was given in the lingual mucosa alongside the frenulum and into the tongue tip.

**Figure 7:**
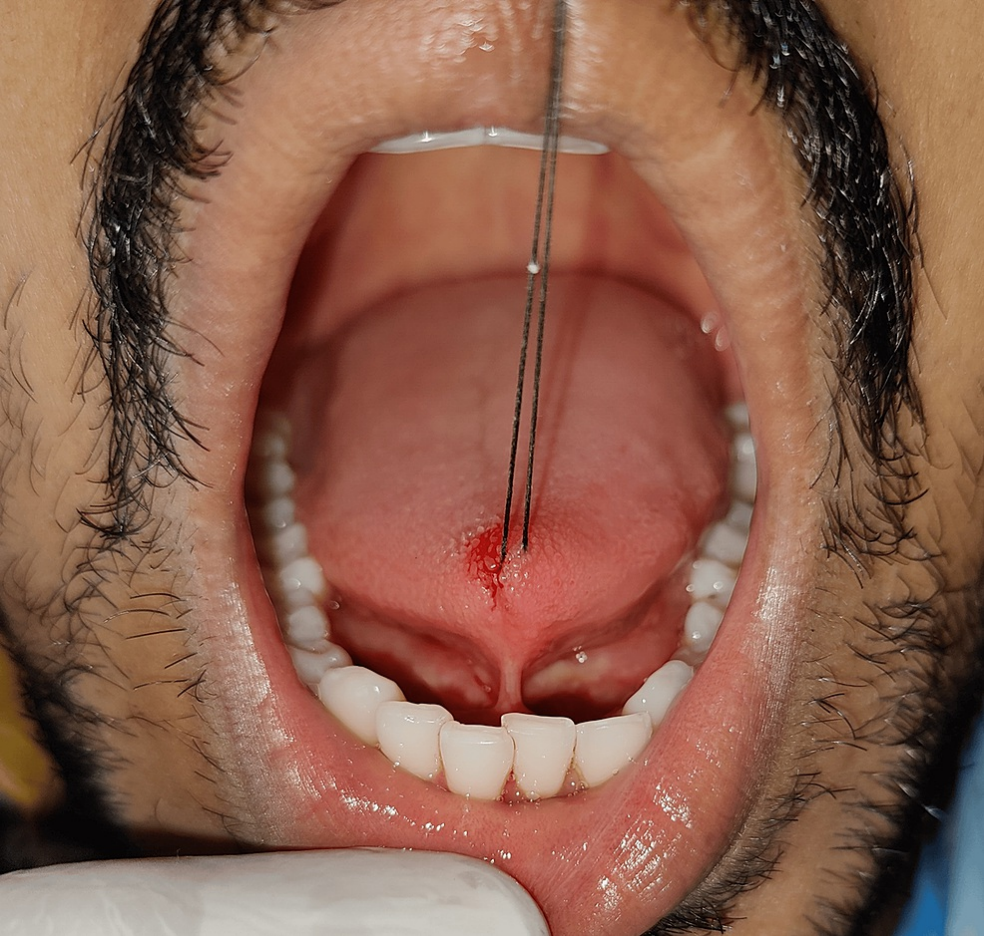
Traction suture at the tip of the tongue

A silk suture was used to stabilize the tongue and was placed at the tongue tip (Figure 7).

**Figure 8:**
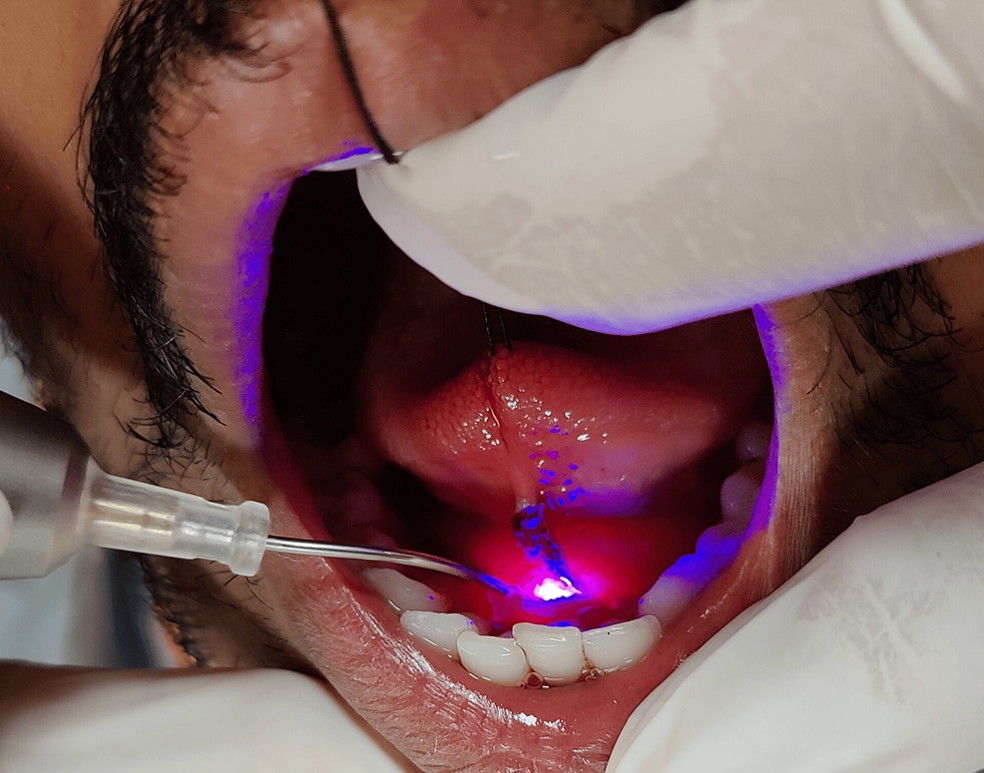
Laser tip initiated by firing; excision of the lingual frenum

A soft tissue diode laser was used to perform lingual frenectomy (Figure 8).

**Figure 9:**
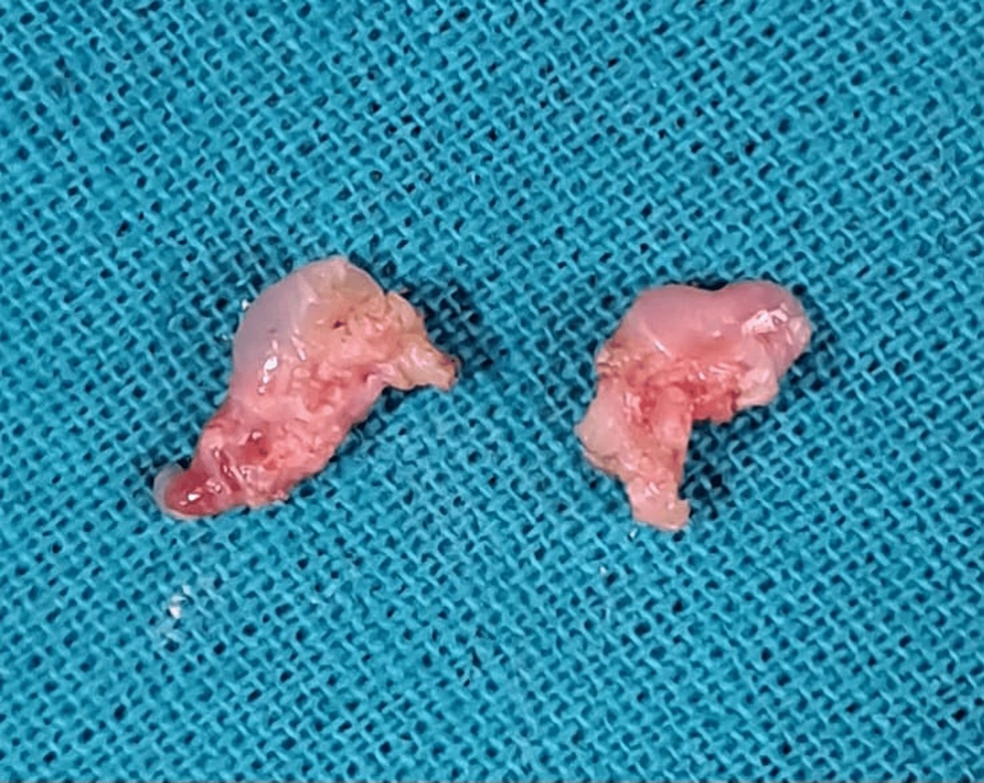
Complete removal of hypertrophic tissue frenulum

A thickened tissue of the lingual frenulum was completely removed, thus preventing its further attachment (Figure 9).

**Figure 10:**
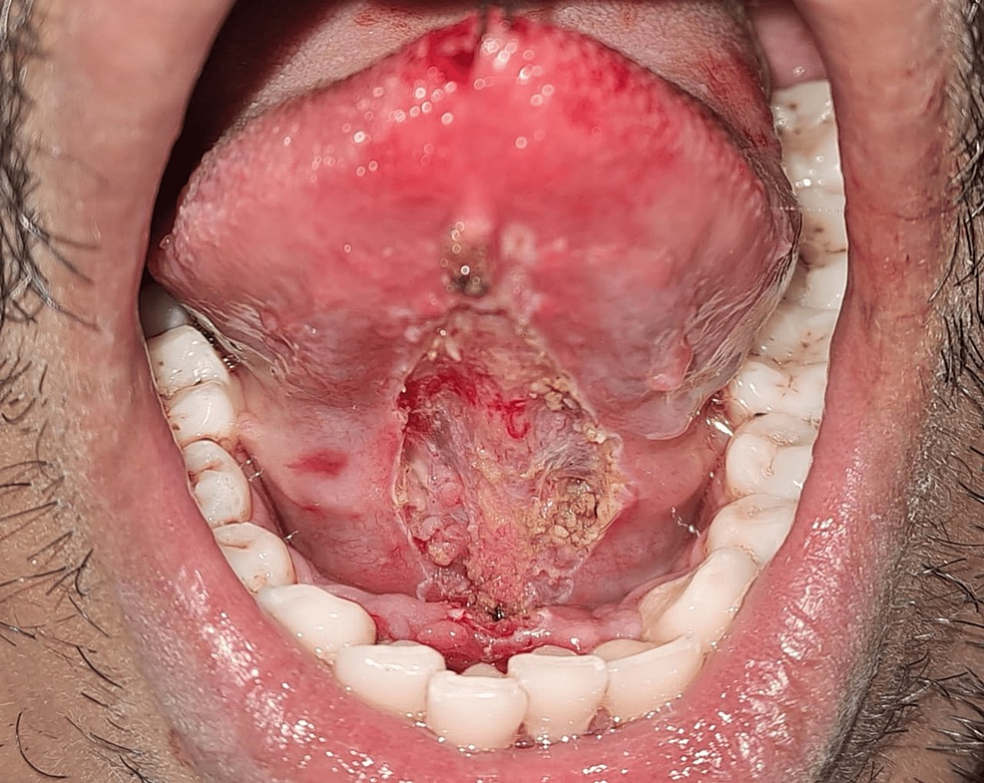
Immediately after frenectomy

After the removal of the thick lingual frenulum (Figure 10).

**Figure 11:**
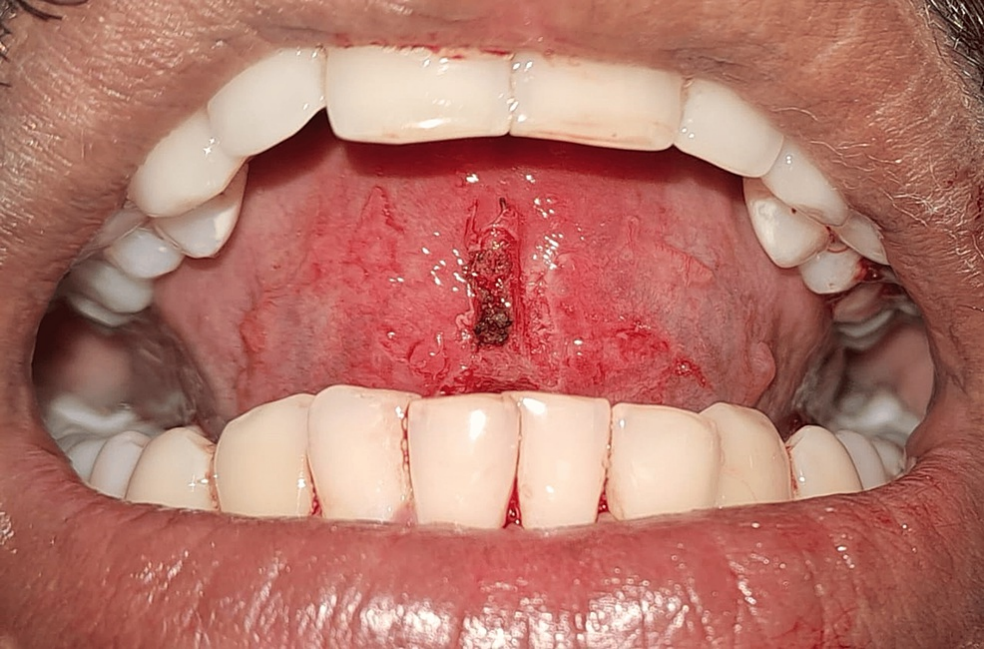
Anterior view after the complete excision of the frenum

The normal saline was used to irrigate the surgical area, and a Betadine gauze piece (Lagaay Medical, Rotterdam, The Netherlands) was placed for 10 minutes under the surgical area to arrest some bleeding points immediately after the complete removal of the lingual frenulum (Figure 11).

**Figure 12:**
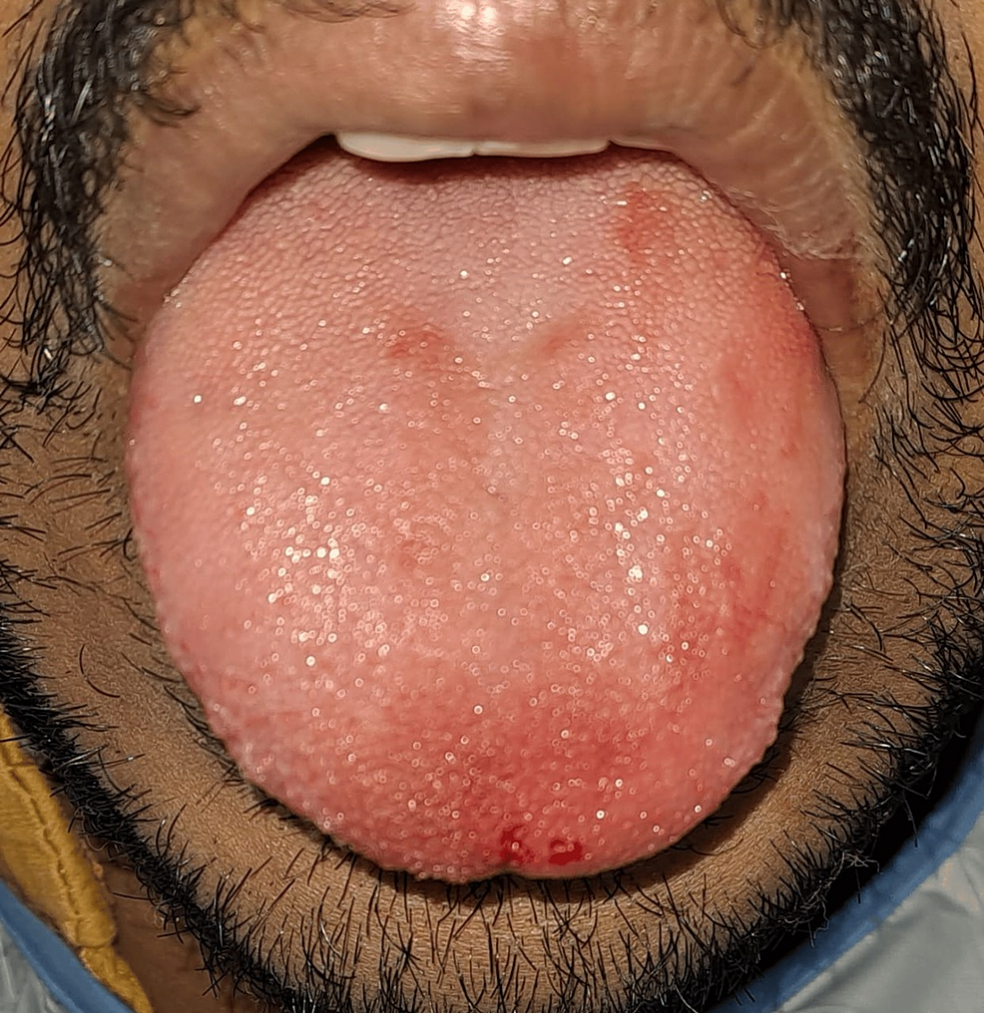
Free mobility and protrusion of tongue after frenectomy

The patient was able to move his tongue freely and had ease in protrusion (Figure 12).

**Figure 13:**
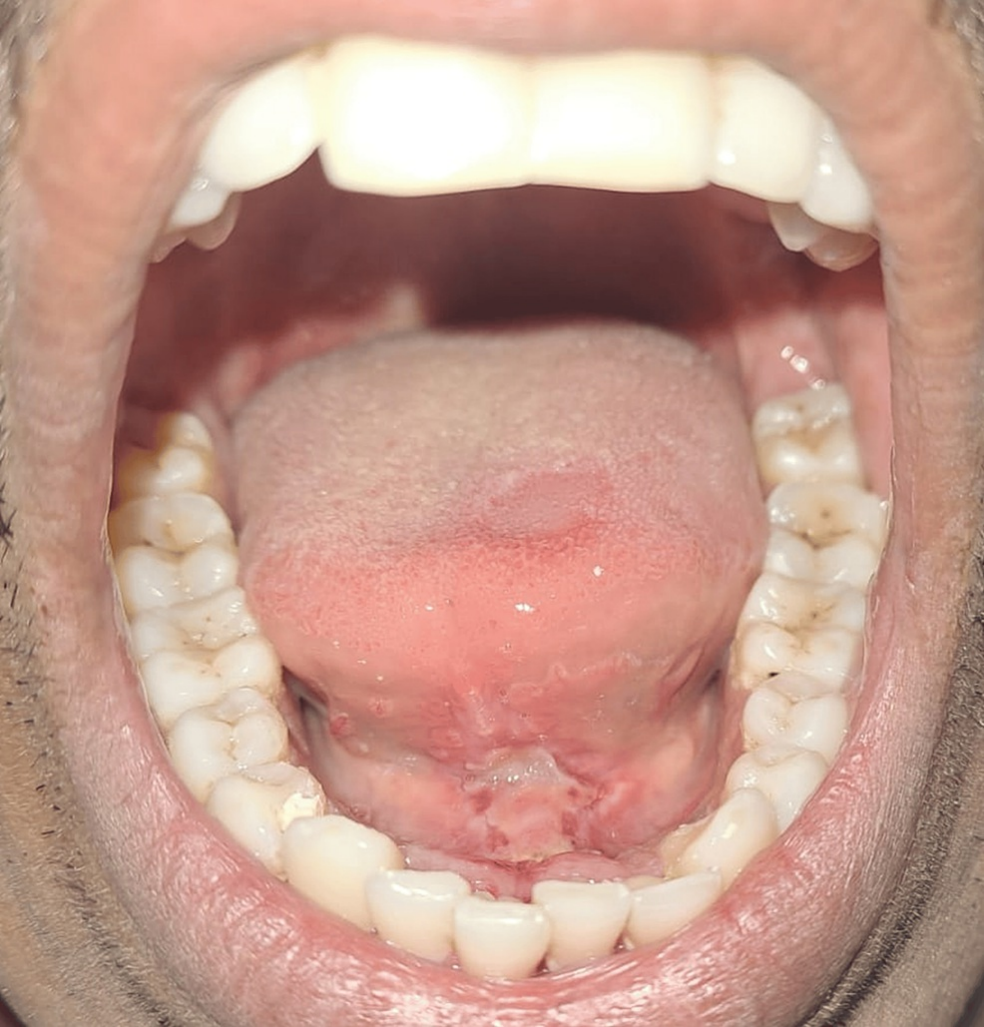
Postoperative follow-up of the patient after one week showing “white soft scab” formation

The patient was instructed postoperatively not to have cold drinks, eat a soft diet, and maintain proper hygiene. Moreover, hot, acidic, and solid food was suspended for the first 24 hours. Patients experienced very minimal difficulty during the first postoperative week, in case of which he is advised to take analgesic tablets for the following five days along with the topical application of gel twice daily. However, partial healing with the formation of a "white soft scab" after the first postoperative week (Figure 13) was noticed.

**Figure 14:**
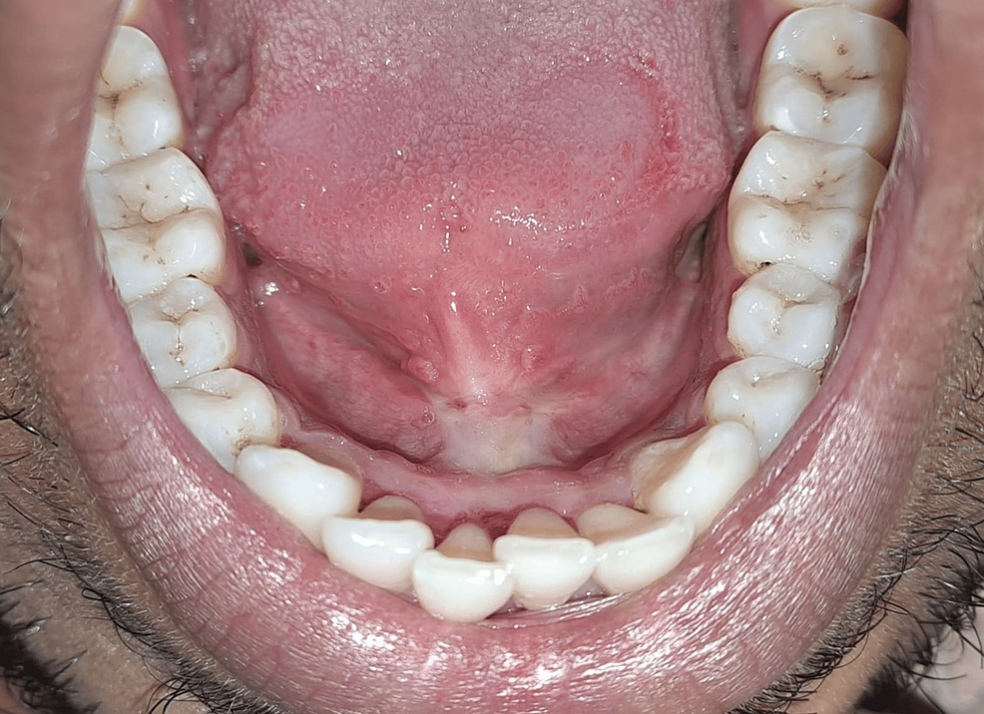
Postoperative follow-up after 15 days, with no scar tissue formation

After 15 days, the surgical area showed satisfactory healing without scar formation (Figure 14).

## Discussion

The demand for laser dentistry is becoming more popular, and it is an alternative to conventional scalpel surgical procedures in the field of dentistry. Many lasers were introduced in dentistry that have been used for frenectomy such as neodymium-doped yttrium aluminum garnet (Nd: YAG) laser, erbium-doped yttrium aluminum garnet laser (Er: YAG laser), diode laser, and diode in conjugation with Er: YAG laser. The establishment of the diode laser was in the mid-1990s [7]. A diode laser is composed of semiconductor crystals of aluminum, iridium, gallium, and arsenic, and it has a built-in solid active medium [8]. The wavelength of diode laser ranges from 445-450 nm, and this low-level wavelength of diode laser shows a high level of the absorption coefficient of pigmented tissue containing hemoglobin, collagen, melanin, and chromophores [9].

Diode lasers are less invasive and provide a bloodless field over surgical areas, with satisfactory healing in the absence of sutures, which lessens the postoperative edema. Low-wavelength diode laser protects the wound from external irritation by molding the protective layer of proteins over the surgical wound. It seals the nerve endings, causing a reduced inflammatory response. Diode lasers circumvent the use of analgesic drugs as they cause less postoperative pain. Hence, diode lasers are becoming more progressive and popular than conventional techniques in dentistry [10]. The primary concept of the diode laser is based on photothermal interaction, making the tissue responsible for the absorption of radiation energy and transforming it into heat energy, which results in changing the structure of the tissues. When the laser makes contact with the tissue (i.e., laser tissue intercourse produces several reactions such as incision, vaporization to coagulation) [11]. For adequacy of the therapy, pain control is quite important for the physical and mental well-being of the patient for routine dental checkups [12]. Laser has the property of sealing the nerve endings, which provides hemostasis, has a coagulation effect on small vessels, and reduces the need for anesthesia [13]. In the present case, the low wavelength of 445 nm diode laser was used for frenectomy, which resulted in no pain or inflammatory edema and gave satisfactory healing without any complications. Anesthesia was infiltrated bilaterally, at the bottom of the tongue before undergoing the procedure. Many studies have advocated the use of a topical spray for anesthesia for performing laser frenectomy. Aldelaimi et al. [14] used midazolam as sedative analgesia along with tropical spray to accomplish frenectomy by laser. In the present case, the patient had good satisfactory healing without any complications and did not notice any kind of postoperative pain, inflammation, or edema. Aldelaimi et al. in their study found that out of 25 patients who underwent laser frenectomy, only two patients experienced postoperative pain, and they were given analgesia for three days [14]. Awooda et al. [15] reported that out of eight patients who underwent laser frenectomy, one patient had persistent symptoms of postoperative pain and another one had developed postoperative symptoms of swelling and pain. Patel et al. [16] and Dixit et al. [17] reported that subjects, which were treated with a diode laser had significantly less postoperative pain and less requirement of analgesia compared with a scalpel procedure.

Laser frenectomy has many advantages over scalpel frenectomy as it is reliable and efficient as a surgical procedure for the soft tissue of the oral cavity. Laser frenectomy can be easily performed with minimal discomfort, great precision, and with no bleeding or minimal bleeding as it seals the blood capillaries by the denaturation of protein and activation of clotting factor VII. A laser wound results in less contraction and scarring as it contains few numbers of myofibroblasts [18]. A disadvantage of laser frenectomy is that it is expensive, creating difficulty in swallowing and poor feeding.

## Conclusions

The patient was very happy with his speech improvement and tongue movement. When the patient came for follow-up after one week, healing with a "rhomboid shape" white soft scab formation was noticed. When the patient again came for follow-up after 15 days, the surgical area showed satisfactory healing without scar formation. The patient was advised to consult with a speech counselor for proper tongue exercises and speech therapy. The case study showed an abnormal insertion of the patient's tongue, which altered its mobility and restrained normal verbal articulation. Rhomboid surgical technique should be applied for the surgical removal of the lingual frenulum to avoid recurrence. Through this procedure and with continuous language therapy, the mobility of the tongue was reestablished, and the patient was instructed to move the tongue as much as possible to avoid coaptation of the sides and undesired adhesions as healing is developed by secondary intention. The use of a low wavelength diode laser becomes relatively complimentary to conventional scalpel lingual frenectomy as it provides better patient comfort, provides a blood-free surgical area, reduces pain, inflammation, and edema, and causes less thermal damage to tissues. Because of its small size, fiber optic delivery, precise cutting, and facileness to operate for oral soft tissue surgery, low wavelength diode laser becomes a choice for a lingual frenectomy.
